# Enhancing the quality of psychological interventions delivered by telephone in mental health services: increasing the likelihood of successful implementation using a theory of change

**DOI:** 10.1186/s12888-023-04829-2

**Published:** 2023-06-06

**Authors:** Cintia L. Faija, Janice Connell, Judith Gellatly, Kelly Rushton, Karina Lovell, Helen Brooks, Christopher Armitage, Peter Bower, Penny Bee

**Affiliations:** 1 School of Health Sciences, Division of Nursing, Midwifery and Social Work, Manchester Academic Health Science Centre, University of Manchester, Manchester, M13 9PL UK; 2 Department of Psychology, University of Sheffield, Sheffield, UK; 3 Greater Manchester Mental Health NHS Foundation Trust, Manchester, UK; 4 Manchester Centre for Health Psychology, Division of Psychology and Mental Health, School of Health Sciences, University of Manchester, Manchester, UK; 5 Manchester University NHS Foundation Trust, Manchester Academic Health Science Centre, Manchester, UK; 6 NIHR Greater Manchester Patient Safety Translational Research Centre, Manchester, UK; 7 NIHR Manchester Biomedical Research Centre, Manchester, UK; 8 Centre for Primary Care and Centre for Health Informatics, NIHR School for Primary Care Research, Manchester Academic Health Science Centre, University of Manchester, Manchester, UK

**Keywords:** Psychological interventions, Telephone, Anxiety, Depression, IAPT, Talking therapies, Feasibility, Implementation, Theory of change, Consolidated framework for implementation research

## Abstract

**Background:**

The implementation of new and complex interventions in mental health settings can be challenging. This paper explores the use of a Theory of Change (ToC) for intervention design and evaluation to increase the likelihood of complex interventions being effective, sustainable, and scalable. Our intervention was developed to enhance the quality of psychological interventions delivered by telephone in primary care mental health services.

**Methods:**

A ToC represents how our designed quality improvement intervention targeting changes at service, practitioner, and patient levels was expected to improve engagement in, and the quality of, telephone-delivered psychological therapies. The intervention was evaluated following implementation in a feasibility study within three NHS Talking Therapies services through a qualitative research design incorporating semi-structured interviews and a focus group with key stakeholders (patients, practitioners, and service leads) (*N* = 15). Data were analysed using the Consolidated Framework for Implementation Research (CFIR) and the ToC was examined and modified accordingly following the findings.

**Results:**

CFIR analysis highlighted a set of challenges encountered during the implementation of our service quality improvement telephone intervention that appeared to have weakened the contribution to the change mechanisms set out by the initial ToC. Findings informed changes to the intervention and refinement of the ToC and are expected to increase the likelihood of successful future implementation in a randomised controlled trial.

**Conclusions:**

Four key recommendations that could help to optimise implementation of a complex intervention involving different key stakeholder groups in any setting were identified. These include: 1-developing a good understanding of the intervention and its value among those receiving the intervention; 2-maximising engagement from key stakeholders; 3-ensuring clear planning and communication of implementation goals; and 4-encouraging the use of strategies to monitor implementation progress.

## Background

Evidence shows psychological interventions delivered by telephone can be as effective as those delivered face-to-face [1, 2], with the added benefits of access, flexibility, and reduced stigma [3–5]. Despite the increased utilization of remote means of communication during the COVID-19 pandemic, some scepticism remains among practitioners and patients about the use of telephone in mental health settings. Concerns centre around the potential difficulties involved in developing a therapeutic alliance at a distance, perceptions of reduced effectiveness, worries about patient safety, and lack of patient engagement [6–8].

The COVID-19 pandemic forced a sudden shift towards remote therapy delivery, with no preparation time in which to train practitioners in telephone-specific skills [6, 9, 10]. The pandemic also drove an increase in mental health problems in the general population in the UK and across the world [11–13]. There were, and still are, pressing needs for mental health services to improve access to psychological therapies without compromising quality of care and to find cost-effective ways of providing evidence-based interventions to an increasingly high volume of patients. In the UK, mild to moderate depression and anxiety are treated in primary care within the NHS Talking Therapies Services (formerly known as Improving Access to Psychological Therapies -IAPT services). Evidence-based interventions are provided following National Institute for Health and Care Excellence (NICE) guidelines. NICE Clinical guidance for mild to moderate depression and anxiety recommends telephone delivery for low intensity psychological interventions such as guided self-help [14, 15]. The use of the telephone as a modality to deliver psychological interventions started in the twentieth century and increased during the recent global Covid-19 pandemic; early indications are that telephone delivery will continue at a comparable, if not greater, pace [16, 17]. This means that the development of a sustainable intervention to improve the quality and engagement of psychological treatment delivered by telephone remains relevant [18, 19].

Before the implementation of any new and complex health care intervention, the Medical Research Council (MRC) framework [20] emphasises an iterative process including four phases: 1-intervention development, 2-feasibility and piloting, 3-evaluation, and 4-implementation. To increase the likelihood of complex interventions being effective, sustainable, and scalable, a theory-driven process of intervention design and evaluation is strongly recommended [21].

The Theory of Change (ToC) approach is a pragmatic framework, which describes how and why an initiative/intervention works and can be useful in both intervention planning and in empirical testing [22]. In addition, the ToC can be subjected to modifications to incorporate ongoing processes of reflection exploring changes and how these happen [23]. According to the MRC framework, a ToC can be tested in the feasibility/piloting phase [20] and its use may help to increase implementation success [24]. This, combined with the involvement of stakeholders, leads to a refinement of the intervention [25]. In this paper, we report on the use of both the ToC and engagement with stakeholders, which strengthen the key stages of the MRC framework [20]. Specifically, a ToC can be modified to capture changes resulting from the feasibility/piloting phase, and a revised ToC can be taken forward for formal evaluation as part of the evaluation phase of the MRC framework. We illustrate the use of the ToC working through an intervention we developed as part of our research programme called EQUITy (Enhancing the quality of psychological interventions delivered by telephone) funded by the UK National Institute for Health Research.

The aim of this article is to explore the use of a Theory of Change (ToC) and an implementation framework of analysis to increase the likelihood of complex interventions being effective, sustainable, and scalable. A ToC was developed to represent how changes of a complex intervention, aimed at enhancing the quality of telephone-delivered psychological therapies, were expected to occur to trigger the intended outcomes. We provide a case example of how the development of a ToC has been informed and amended following evidence from the feasibility study reported here. We also describe potential solutions to overcome implementation challenges and improve likelihood of future implementation success.

## Method

### Description of the EQUITy intervention

The EQUITy intervention was a service quality improvement intervention designed to enhance engagement and the quality of psychological interventions delivered by telephone in primary care in the UK. In brief, the EQUITy intervention targets change at service, practitioner, and patient levels and includes three interlinked components that are equally important: 1-Telephone Recommendations for Services, 2-Telephone Skills Training for Practitioners and, 3-Telephone Information Resources for Patients.

The Telephone Recommendations for Services comprised a booklet including guidelines for telephone delivery covering five areas (i.e., Promoting Telephone Work, Key Elements of Telephone Work, Telephone Working Environment and Resources, Boosting Telephone Skills, and Promoting Reflection on Telephone Treatment). Services were asked to decide on a set of goals that could be achieved immediately, and another set that may take longer or need more effort to accomplish. This booklet was circulated to the Service Lead/Team Manager. This component of the EQUITy intervention was designed to ensure availability of the most suitable working environment and resources for the delivery of psychological interventions by telephone, enhance the provision of clinical support for remote delivery and facilitate opportunities for professional development.

The Telephone Skills Training for Practitioners included two 3-h online training sessions, combining teaching and interactive activities (e.g., role-play, live demonstration of good practice, group exercises). This component of the EQUITy intervention was designed to develop and/or enhance practitioner telephone skills, address negative preconceptions, and increase engagement and confidence in the delivery of psychological interventions by telephone.

The Telephone Information Resources for Patients comprised a leaflet containing key information about telephone interventions delivered by psychological well-being-practitioners, an appointment card which also included tips on how to prepare for telephone sessions, and a poster to be displayed at services. This element of the EQUITy intervention was designed to increase awareness of cognitive behavioural therapy and guided-self-help, and its effectiveness when delivered via telephone; it also aimed to address beliefs and pre-conceptions about remote delivery being inferior in quality to face-to-face delivery.

The EQUITy intervention was delivered by a team comprising the two principal investigators (PBo, PBe), the programme manager (JG), a clinical academic with robust experience of delivering training and providing therapy as a Cognitive Behavioural Therapist (KL), a clinical psychologist, two psychological wellbeing practitioners, one clinical academic involved in delivering training for PWPs, one researcher with clinical experience in different evidenced-based therapies including Cognitive Behavioural Therapy (CF) and one patient with experience in supporting training of psychological wellbeing practitioners.

### The use of ToC exemplified in the EQUITy Programme

- **(a) Aim of using ToC model**

**Fig. 1:**
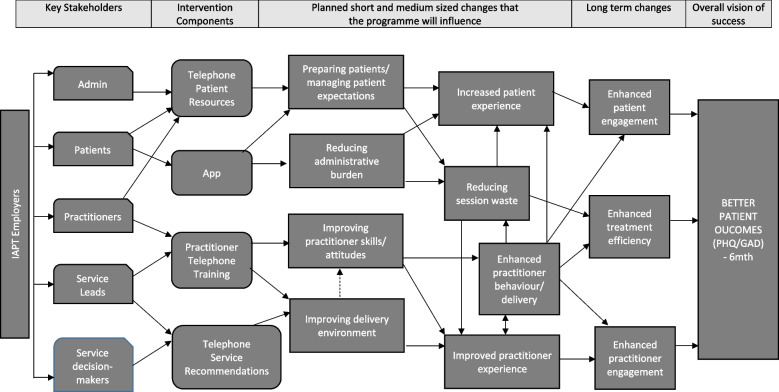
EQUITy Programme: Initial Theory of Change Note: The dash line indicates weaker pathways/linkages

A ToC was developed for the EQUITy research programme to represent what the intervention intended to achieve and to depict the most likely linkages/pathways of how changes were expected to occur/trigger the intended outcomes. To this end, key stakeholders, objectives, intervention components, intended changes/goals (short, medium, and long-term), and an overall vision of success were identified beforehand (see Fig. 1 for a schematic depiction of the EQUITy initial ToC). As depicted in Fig. 1, the short/intermediate goals of the EQUITy intervention were to enhance patient, practitioner, and service engagement with telephone treatment, leading to increased treatment efficacy and effectiveness, and to ultimately improve patient outcomes (i.e., symptoms of depression and anxiety over time).

- **(b) Who was involved in the initial development of the ToC?**

Six members of the research team with extensive experience in the use of the ToC and implementation research reviewed qualitative evidence to inform intervention design and development of the initial ToC.

- **(c) Method by which the ToC was developed**

The initial ToC was developed by reflecting upon the qualitative evidence obtained from research studies conducted within this research project, which provided an in-depth understanding of the clinical and organisational contexts in which telephone-delivered psychological interventions are used. Qualitative studies included interviews with practitioners, patients, and key informants, as well as the conversational analysis of telephone sessions delivered in IAPT services. Evidence from the qualitative studies provided an insight into the barriers and facilitators influencing delivery and engagement of patients, practitioners and services with psychological interventions delivered by telephone [6, 7, 26–28]. Following understanding of the problem, an evidence-based behaviour change intervention was co-developed with stakeholders to increase engagement and quality of telephone-delivered psychological interventions in primary care [29]. In addition, a smartphone app was developed to facilitate completion of questionnaires and to improve therapeutic information exchange in telephone-delivered psychological interventions.

- **(d) Who was involved in reviewing/refining the ToC**

**Fig. 2:**
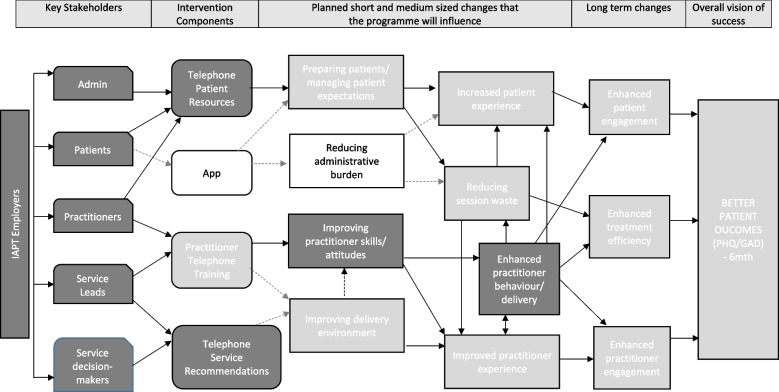
EQUITy Programme: Theory of Change updated reflecting the COVID context (trialled in this feasibility study) Note: White boxes indicate elements removed from the intervention prior to the commencement of the feasibility study. Light grey boxes highlight elements that are potentially diluted/uncertain and indicate additional uncertainty/risk. Grey dash lines indicate diluted pathways/linkages

The initial ToC (Fig. 1) was reviewed by the research team to reflect the possible impact of COVID on the model, producing a modified ToC (Fig. 2) which was trialled in this feasibility study. Figure 2 indicates that the smartphone app was removed from the EQUITy intervention due to it being deemed redundant following a shift by services towards the use of online portals for outcome measure completion and communication with patients. In addition, Fig. 2 captured elements of the EQUITy service quality improvement intervention that could be potentially diluted/influenced by the fact that services were forced to deliver psychological treatment by telephone (or video) during the pandemic, which may have also affected patient views/expectations about telephone treatment.

Following findings from this feasibility study, a meeting with all members of the research team was held to interrogate and review the ToC for appropriateness, comprehensiveness and accuracy. This included the identification of components and theoretical changes that were achieved or not met, and the examination of whether or not and how the ToC aligned with the findings. Identified changes to the components of the intervention and ToC were then further discussed with the Implementation Reference Group (IRG) and the Programme Steering Committee (PSC). The IRG comprises10 professionals with clinical, policy or educational expertise in IAPT services to provide strategic advice and guidance to the design and the implementation of the EQUITy programme. The PSC is an independent group of five individuals with research and clinical expertise in low intensity psychological interventions, service organisation, delivery, development, and leadership. One member has specific expertise in the use of statistics in randomised controlled trials, while a patient and public representative provides additional expertise from a lived experience perspective. Following agreement, the required amendments to the ToC and the intervention took place. The updated version of the ToC will be evaluated as part of a randomised controlled trial.

### Ethical approval

Ethical approval for the EQUITy feasibility study was granted by the North West—Preston Research Ethics Committee (REF: 20/NW/0082; IRAS ID: 271710cif). All participants completed the consent form, which they returned via email.

### Study design & recruitment

This was a qualitative study using semi-structured interviews and a focus group with stakeholders (patients, practitioners, and service leads). Interviews [30] and focus groups [31] were used to account for service preferences and convenience, and to allow exchange of viewpoints whenever this was considered appropriate. The epistemological position underpinning this study followed a social constructionist approach [32–34].

This feasibility study was conducted in three UK NHS IAPT services (currently known as Talking Therapies services) delivering guided-self-help for anxiety and/or depression by telephone. NHS IAPT services were approached via email direct invitation to service managers prior to the outbreak of the COVID-19 pandemic, and their commitment remained during the pandemic, when the research study commenced.

### Stakeholder informants: patients and professionals

Patients receiving guided-self-help for anxiety and/or depression by telephone at the services participating in the study were invited to take part in a research interview. Information packs containing an invitation letter, participant information sheet, and a consent to contact form were distributed by the service administration team. Following expressions of interest (return of consent to contact form or direct contact with the research team by email or phone), participants were contacted to confirm willingness to participate, complete the consent form and to schedule a telephone interview. Examples of interview questions for patients included: What did you know about IAPT when you were first referred? What was the information you received at the point of referral and what can you tell me about that information? What did you think about the EQUITy patient resources? How did the EQUITy resources influence (or not) your views about the effectiveness of the treatment you were referred to? How did the EQUITy resources change (or not) your beliefs about how you will be able to successfully engage in your treatment? Thinking about your experience of treatment, how did you find communicating with you practitioner without being able to see her/him? What can you tell me about the therapeutic relationship with your practitioner?

Professionals, including practitioners and service leads from each of the three services, were invited to take part in a research interview/focus group. Practitioners delivering guided-self-help by telephone who had attended the EQUITy telephone skills training were invited to take part in the study. All eligible professionals received an invitation letter, a participant information sheet, and a consent to contact form by email, distributed by the service lead/manager or research team. Participants expressing interest to take part were contacted by the research team to confirm willingness to participate and an interview/focus group date was arranged. Examples of interview questions for professionals included: What were your initial thoughts about the EQUITy intervention and taking part in the feasibility study when you heard about it? What aspects of the EQUITy intervention were most helpful for patients, practitioners and for services? What aspects of the EQUITy intervention would you change? How did you feel about delivering telephone treatment following receipt of the EQUITy intervention? What were the challenges you face to implementing the EQUITy intervention? What has been the most significant change in your practice following the reception of the EQUITy intervention?

### Data collection

The study took place in England, UK. The EQUITy intervention was delivered to each of the three NHS IAPT services independently between November 2020 and January 2021. Data collection took place post-intervention between February and April 2021.

All participants provided informed consent prior to taking part in the study. All interviews/focus group were audio-recorded and transcribed verbatim by an independent company approved by the University of Manchester. Any identifiable information was removed from the transcripts to protect participants’ anonymity and data were securely stored.

Fifteen stakeholder informants across the three NHS services took part in the study (i.e., six patients, five practitioners, and four service leads). All six patient participants had received two or more sessions of guided-self-help by telephone from a practitioner who had received the EQUITy telephone skills training. All patient participants were females, aged between 20 to 57 years old (M=35.33, SD=15.67), five self-described as White and one as Asian, four were employed full-time, one was not employed due to health issues, and one was retired. Three patient participants were receiving treatment for anxiety and depression and the rest for depression only. Three had completed telephone treatment at the time of the interview and the other three were still in treatment.

The professional participants included five practitioners with varying degrees of experience from trainees to senior practitioners with supervisory responsibilities, and four service leads. Eight out of the nine professionals were female, all were White British, aged between 24 and 59 years old (M=36.44, SD=11.46). The professionals’ experience within mental health ranged from more than one year up to 20 years.

All participants were interviewed and two service leads from one NHS IAPT service participated in a focus group. A focus group was particularly pertinent as they were undergoing a service restructure at the time, enabling a more meaningful and fuller discussion about service implementation . The interviews and the focus group were conducted by telephone and lasted between 40 and 69 minutes.

### Data analysis

Data were analysed incorporating inductive and deductive approaches with deductive approaches informed by the CFIR framework [35]. The CFIR is a widely used pragmatic multi-level framework that provides an organized list of potential barriers and facilitators to assess the implementation of complex interventions. It is composed of a taxonomy of 39 operationally-defined constructs across five major domains: (1) intervention characteristics, (2) outer setting, (3) inner setting, (4) characteristics of the individuals involved, and (5) the process of implementation. Each major domain includes several sub-domains. We used the CFIR to identify the areas that are most important to optimise our intervention and subsequently to explore how these data could be used to inform the development of a ToC.

Firstly, researchers familiarised themselves with the transcripts and the CFIR framework. Secondly, data were coded into the corresponding domains/sub-domains of the CFIR framework and data outside of the framework were coded inductively. CF coded service leads’ data and JC coded data from patients and practitioners. Thirdly, findings were regularly presented and discussed with the wider research team to facilitate further reflection and inform the changes (if needed) to the ToC. The EQUITy intervention was a service quality improvement intervention and targeted change at three different levels (patients, practitioners and service leads); however, the immediate recipients of the intervention were practitioners and services. Consequently, the ToC was used to explore implementation barriers and facilitators, and the patient data aided understanding of the reach of the intervention. Data analysis was supported by QSR International’s NVivo-12 qualitative software [36].

## Results

Table 1 presents an overview of the five CFIR domains and associated sub-domains that were relevant for the implementation of our service quality improvement intervention and it also provides detail on the key challenges identified during implementation. In this section, we provide a summary of the CFIR findings and include participants’ quotes throughout.

**Table 1:** CFIR domains and sub-domains, and key challenges identified during implementation

*Domain*	*Sub-domain*	*Key challenges*
**1-Innovation Characteristics**	**Relative Advantage**	Emphasising the additional value of the intervention
		Increasing awareness of the multiple components of a service quality improvement intervention
	**Design Quality & Packaging**	Developing user-friendly and easily accessible materials
**2-Outer Setting**	**Patient Needs & Resources**	Patient information: effective communication routes and friendly easy-to-read information
	**External policy and incentives**	Increasing use of remote delivery of psychological interventions
**3-Inner setting**	**Structural Characteristics**	Staff turnover
	**Implementation Climate (tension for change, compatibility, relative priority and goals and feedback)**	Intervention highly compatible but of low relative priority and lack of impetus for change
		Clarity on implementation goals and feedback
		Using incentives and rewards
	**Readiness for implementation (leadership engagement, resources available, and access to knowledge and information)**	Leadership engagement
		Organizational resources
		Awareness and knowledge
**4-Characteristics of individuals**	**Knowledge and beliefs about the intervention**	Beliefs, attitudes, and value placed on the intervention
		Factors influencing a positive telephone treatment experience
**5-Process**	**Planning**	Formal planning
	**Engaging**	Engagement of appropriate individuals

### 1-Innovation characteristics

We described three key points relating to the sub-domains of relative advantage, and design quality and packaging that may weaken successful implementation of our service quality improvement intervention.

- ***Emphasising the additional value of the intervention***

Illuminating stakeholders’ perception of the advantages of an intervention and the degree to which it can be adapted and tailored to meet service needs are important factors to address prior to its implementation. Due to the COVID-19 pandemic, IAPT services had increased the delivery of psychological interventions by telephone at the time the EQUITy intervention was introduced. The lack of clarity about the potential benefits of its use and its addition to current practices at each service may have weakened professionals’ motivation towards its implementation. Indeed, service leads and practitioners shared these views, as illustrated in the findings below:

*“So I think, perhaps, if it wasn’t as established, it would have been really useful to have, you know, a Zoom meeting, initially. What’s it about, what would be useful, what can we get from it”. (Service Lead 4).*

*“I’d say just discussing telephone working as a whole, because I’d say with the university course, you’re taught to deliver the interventions and taught how to conduct an assessment, you’re not necessarily taught how to develop those telephone skills”. (Practitioner 6).*

- ***Increasing awareness of the multiple components of a service quality improvement intervention***

We found that if a service quality intervention includes multiple components and requires engagement from different stakeholders, providing stakeholders with a clear presentation reflecting the integration of and rationale for the different elements is extremely important in improving the likelihood of implementation success. For instance, the EQUITy intervention involved three elements (i.e., Telephone Recommendations for Services, Skills Telephone Training for Practitioners and Telephone Information Resources for Patients), but service leads and practitioners perceived it as a telephone training intervention instead of a service quality improvement intervention. Findings highlighted there was a lack of awareness from practitioners of two of the three elements of the intervention (i.e., Telephone Recommendations for Services and Telephone Information Resources for Patients) and service leads placed more value and importance on the Telephone Training for Practitioners. Below is a quote from a dialogue with a practitioner and one with a service lead illustrating these findings:

*Participant: “I don’t even know where that service recommendations book is, if we’ve had it even.(Practitioner 1)*

*Interviewer: “Right, so you have no knowledge of that at all?” *

*Participant: “No, not seen it, I’ve not seen the booklet, so I don’t know. I honestly don’t know if we’ve had them”. (Practitioner 1)*

*Interviewer: “…what were your initial thoughts about the EQUITy intervention and taking part in the feasibility study when you heard about it?”*

*Participant: “So, is this just the sort of telephone training?” (Service Lead 3)*

Consequently, the implementation of the Telephone Recommendations for Services and Telephone Information Resources for Patients components was compromised, weakening their contribution to the change mechanisms set out in the ToC.

- ***Developing user-friendly and easily accessible materials***

We found that when a service quality intervention targets different key stakeholder groups, the design of materials should be user-friendly but also easily accessible, facilitating communication with members of the team, encouraging participation, ensuring good flow, and allowing editing from different contributors (e.g., service leads, practitioners, and administrative staff). Below is a quote from a service lead illustrating the challenges faced in populating one of the documents provided as part of the intervention:

*“[referring to the Telephone Guidelines for Services] I just remember, when I, I took the task on, and I sort of identified the things that we already do, and some that we could perhaps do better, I just remember, when I was trying to use it, to write on it, you know, it was hard to do, and I can't remember exactly why, now. But it wasn’t an easy document to insert information into”. (Service Lead 4)*

### 2-Outer setting

All mental health services were tasked with increasing remote modalities of working, particularly telephone, during the COVID-19 pandemic. The challenge faced was how to deliver telephone treatments as effectively as those delivered face-to-face. Within the outer setting domain, we found that sub-domains of patient needs and resources and external policy and incentives were the most relevant for our quality service improvement intervention. We outlined two key points that may weaken successful implementation.

- ***Patient information: effective communication routes and friendly easy-to-read information***

We found it was important to explore effective routes to deliver patient information to prevent resources from getting lost among other materials routinely received by patients from mental health services. Since COVID, most services use email to communicate with patients, but most patients taking part in this study could not recall receiving our Telephone Information Resources for Patients. One remembered receiving the EQUITy patient leaflet and said:

*“So I remember it [patient leaflet] being quite simple, quite easy to look at and didn’t make it feel like a big deal, which is very nice […] so it can feel very kind of, yeah, overwhelming, whereas something that’s sort of short and simple and not intimidating is really helpful”. (Patient 1)*

Patients accessing mental health services appreciated the difficulty of finding the balance between providing a lot of information without this being overwhelming. Some found the information provided by services in general was easy to read and to understand and was sufficient for their needs. Others, however, pointed out that grasping it was much more difficult when feeling unwell and valued information that was presented clearly and simply in a user-friendly manner. This finding became evident from the patients’ data but was also shared by practitioners and service leads. In addition, one service lead highlighted the importance of going through the information with the patient during their first appointment:

*“And the importance of, I guess, that the practitioner is introducing it to them, and it's not just something that’s sent to them which they might just overlook”. (Service Lead 4)*

Patients, practitioners, and services leads thought that text-heavy documents could be broken down into sections and the use of colour and diagrams could help make the information less intimidating. A colourful leaflet, such as the one provided as part of the EQUITy intervention, softened the impact of the stark impersonal nature of the NHS appointment letter.

*“I just really liked the layout of it, I think the colours are really helpful to break it up. I think even though there is a lot of information on there, I do think it helps splitting them up into different text boxes and the bright colours that you’ve used, I think that does help”. (Practitioner 6)*

- ***Increasing use of remote delivery of psychological interventions***

Findings from service leads and practitioners highlighted that the provision of remote delivery of psychological treatments had increased due to COVID. Prior to COVID, the use of telephone was different across sites, ranging from rarely using telephone to delivering most of the assessments and treatment sessions by this modality at Step 2 care (i.e. guided-self-help) only. Practitioners and service leads anticipated that the vast bulk of work would continue via telephone (and/or video) due to its proven effectiveness:

*“There are no noises yet that they’re going back face to face, that if they’re working digitally, we’re going to keep it going that way. So I think there will be more phone and more video”. (Practitioner 5)*

*“But now it’s 90 per cent telephone and about ten per cent online … But you don’t need all the PWPs being able to see face to face, they should have an allocated, maybe one or two that will pick up those and everyone else carries on, on the phone. So they don’t need to see people, because this has worked well, this works really well”. (Practitioner 1)*

*“So definitely promoting that, because I think after COVID, the majority will still be telephone work as well, so we’ll have to keep that up and promoting it”. (Service Lead 1A)*

Service leads indicated costs will be reduced due to not needing physical space to deliver treatments face-to-face, which is the main driver for more treatments being delivered remotely:

*“But I still think that the vast bulk of work will be telephone, and even, sort of, after COVID, you know, we’re looking at giving up clinic rooms because that’s going to be a saving to the service. And really, kind of, reducing that down, so where a PWP in the past might have had two full days in clinic, reducing that down to, sort of, half a day and the rest of that time being done, sort of, over the telephone, via Silver Cloud or via video”. (Service Lead 3)*

This indicates the relevance of this topic and the importance of ensuring successful implementation of interventions designed to improve the quality of telephone working.

### 3-Inner setting

The sub-domains of structural characteristics and implementation climate, including tension for change, compatibility, relative priority, and goals and feedback, were the most relevant. In addition, sub-domains related to readiness for implementation such as leadership engagement, resources available and access to knowledge and information were key to understanding organizational commitment to implement a service quality improvement intervention, and its incorporation into routine practice. We described seven aspects that may weaken successful implementation.

- ***Staff turnover***

Mental health services are stretched and face high staff turnover and if no strategies are in place to engage relevant stakeholders, the incorporation of the intervention into daily practice becomes compromised. For instance, our intervention required practitioners to attend two 3-h online telephone skills training sessions. If attendance is low due to service clinical commitments and/or those attending the training subsequently leave the service, are redeployed or are on sick leave, the implementation of the intervention is at risk of failure:

*“I think once we had the training very shortly after that, this [COVID] all started, and I think everybody’s just…because some people have been redeployed, people have been moved left, right and centre, so we’ve not really had much stability”. (Practitioner 1)*

*“Over the last 12 months when people are getting stressed or they are getting burnt out, that seems to be the sort of, common theme that’s running through”. (Service Lead 3*)

To increase the likelihood of success, knowledge gained by practitioners attending the training should be cascaded down to other colleagues and to the service, to ensure sustainability over time.

*“If some of the staff are part of that initial training, then that will embed it again. So especially if they’re supervisors, because then they are going, it will percolate down to their supervisees, as well. So, in that way, it’s to keep them advised”. (Service Lead 4)*

It is anticipated that the development of strategies to immerse staff not directly engaged with the intervention, i.e. new staff or those unable to attend initially, will be pivotal in ensuring their understanding of the intervention and in identifying their role in its implementation and sustainability in their service.

*“But also, we were talking about, when our new staff are coming in – we've got 18 PWPs coming in, in March – having a training session for them, solely on telephone work.” (Service Lead 4)*

Access to telephone training resources online could prove beneficial to overcome the problem of staff turnover:

*“I think, if it was almost like a package that was available to almost have on video, sort of thing, you’ve got that, you know, as staff teams change which they do so frequently, it’s there, it’s readily available, people can access it, people can go on refreshers. I’m thinking, you know, I’ve just had someone come back off six months off on sick leave, and they, sort of, feel that they don’t know anything”. (Service Lead 3)*

- ***Intervention highly compatible but of low relative priority and lack of impetus for change***

All services were already using the telephone as a treatment modality when our intervention was introduced, and most services envisage that the increased use of the telephone because of the COVID-19 pandemic will continue. Therefore, improving the quality of telephone treatment for patients, professionals, and services was identified as an area of high priority and interest. Data from professionals highlighted the importance of tailoring service quality improvement interventions for each service, and for its workforce, to guarantee success. For instance, the telephone skills training element of our intervention was reported to be more useful for recently qualified professionals than for those with a few years of experience using the telephone. Nevertheless, the most experienced practitioners highlighted that their confidence in current practice increased, and they had developed new skills and broadened their knowledge. They had also benefitted from reflections and discussions about how to overcome challenging scenarios when using the telephone and it had been a good opportunity to identify “bad habits”:

*“I think for me, I’ve been doing it that bloody long now, it’s kind of…it’s not a bad thing to kind of reflect on it and look at bad habits, a lot of it. So although I’m confident in doing phone stuff, because when I did my training, my service literally did all phone, so it’s kind of always been the norm for me. I’m more confident on the phone than I am face to face, weirdly. I just like to be different. So I’ve been doing it that long that learning a bit of theory and stuff is probably a good thing.” (Practitioner 5)*

Telephone practices in the services taking part in the EQUITy feasibility study were not perceived as needing a radical change, and in combination with clinical demands and busy workloads, the implementation of the intervention was not identified as a service priority; it is hypothesised this compromised motivation and engagement towards its implementation:

*“we’re all just struggling to do our basic job as it is and I think anything extra they just don’t want anything else more than they have to deal with. It’s not been pressed as a priority at the moment I think that might be what it is.” (Practitioner 1)*

*“I think, real life in some ways took over and they couldn’t make it a priority for them”. (Service Lead 3)*

- ***Clarity on implementation goals and feedback***

Professionals revealed that implementation goals and strategies to monitor the progress of the implementation of the intervention were not in place; and service leads did not report on providing feedback to their practitioners about it. Clear communication and monitoring processes from service leads and/or team managers relating to the implementation of the different elements of the intervention set from the start may prove fruitful in increasing implementation success.

“*I know nobody’s raised it, or had a conversation about it, so it’s just fell by the side”. (Practitioner 1).*

*“I don’t think we’ve been made aware of anything else with regards to this study at the moment”. (Practitioner 3)*

*“So just getting those specifics from us of what we’re going to implement first from the recommendations. And maybe could do a follow-up to see if those things have happened.” (Service Lead 1B).*

- ***Using incentives and rewards***

Findings highlighted there was an absence of the use of incentives and rewards to foster implementation. One of the practitioners reported that accrediting the EQUITy training as part of a CPD course could be perceived as a good incentive.

*“I suppose the only thing that I can think of really is if it was, for instance…because hopefully, as PWPs moving forward, we will get some accreditation maybe by the BPS. I don’t think we’ll get it *via* the BABCP, but potentially… I don’t know whether it’s like they will have like a… Not exactly a CPD certificate, but something that would count towards like CPD or something that they can get out of it, if it makes sense. Not exactly like a little reward but some sort of incentive, if that makes sense”. (Practitioner 4)*

- ***Leadership engagement***

Leadership engagement with and the accountability taken by leaders and managers for the service quality improvement intervention influenced the success of its implementation. Service leads across sites engaged at different levels with the implementation of each of the three different elements of the intervention. For instance, one lead attended the telephone skills training for practitioners but did not engage with the service booklet. Leads from the other two services did not attend the telephone skills training but one set the training as mandatory for their staff, and both services paid close attention to the Telephone Recommendations for Services and made changes accordingly (details about the implemented changes are reported under the “process” domain). All service leads reported that telephone information resources for patients were delivered by email but the majority of patients could not recall receiving them.

- ***Organizational resources***

All services reported busy workloads and limited availability of internal resources to receive and implement the intervention, including limited time for staff to attend training sessions due to clinical commitments and subsequent lack of time to discuss implementation and monitor its progress:

*“I know that you initially said that we could have it for the whole service, for the whole of the PWPs, but because of obviously targets and contacts and them taking a day out, we weren’t able to give it to all of the PWPs, which is a shame really.” (Service Lead 1A)*

*“So, you know, from a sort of, capacity point of view we don’t have time to, sort of, send the whole team on this training”. (Service Lead 3)*

*“You get the contacts taken off, your daily contacts, but your caseload doesn’t change, so you still have to fit those clients in somewhere and it’s where it becomes a bit tricky.” (Practitioner 3)*

*“For PWPs, usually the reason for doing anything or not doing anything is time, that you just get so caught up in the ever increasing list of treatments that you’re doing and assessments that it does feel like there’s no time for anything else.” (Practitioner 5)*

Early discussion and planning around how to incorporate the intervention within existing procedures and available resources could potentially facilitate successful implementation.

- ***Awareness and knowledge***

A lack of awareness and knowledge about two of the intervention components (i.e. Telephone Recommendations for Services and Telephone Information resources for patients) and how to incorporate each of them into day-to-day work tasks was found in the practitioners’ data:

*“I don’t really know. I just thought that we attended the training and then we were having this sort of interview with yourself and that was really all we had to do. I never really picked up on anything else.” (Practitioner 3)*

Data have shown there was confusion about who was responsible for distributing the resources to patients, and with respect to when (e.g., before or after the patient assessment appointment) and how (e.g., post or email) they should be distributed. It is hypothesised that greater clarity among team members involved in the intervention around how to implement it in their day-to-day work (i.e. what is needed and from whom) could prove beneficial.

*“We have received the leaflets, but we haven’t been told what to do with them”. (Practitioner 1)*

### 4-Characteristics of individuals

The sub-domains of knowledge and beliefs about the intervention provided key information to further understand what requires prioritisation to optimise its implementation. We hypothesised that self-efficacy and individual stage of change sub-domains might not appear to be relevant due to the lack of clarity around how to implement each element of the intervention into daily practice and as a result or regular telephone use during COVID. Below we described two key aspects within this CFIR domain.

- ***Beliefs, attitudes and value placed on the intervention***

Professionals’ beliefs and the value they placed on the intervention are key drivers towards the success of its implementation:

*“I think my initial thoughts were actually, this is a really good project to be involved in and something that I personally would love to learn more about. Because I’ve…again I’ve done it for years and years and never felt trained in, sort of, delivering treatment over the phone.” (Service Lead 3).*

Individual beliefs about personal capabilities to apply the intervention into routine practice and individuals’ motivation to change their current way of working over the telephone are important in reducing its potential failure. For instance, practitioners who were aware of the patient telephone information leaflet reported this was useful but there were concerns and a reluctance to use it due to fear of overburdening patients. Thus, identifying and challenging these beliefs early on during the implementation phase may remove these barriers to implementation.

The majority of practitioners reported the importance of allocating more time during the telephone training to practise skills and indicated it would be beneficial to incorporate into the training specific psychological interventions such as behavioural activation, or exposure and its associated challenges when using this modality.

- ***Factors influencing a positive telephone treatment experience***

Findings from patient interview data provided information about key factors that contribute to a positive telephone treatment experience, including the development of a good therapeutic relationship, receiving personalised care, and feeling listened to by their practitioner.

The skills aiding a good therapeutic relationship reported by the patients were: going at a pace that suited them; guiding things along without pushing; keeping focused; listening and responding; being patient and understanding; being genuinely caring and empathetic; providing reassurance; being informal, warm, and friendly.

Patient interview findings showed practitioners cared about them via listening carefully, using their verbal skills such as by having a soothing voice, remembering and recapping what they had said in previous sessions, checking they were ok, and using appropriate utterances and changing tone of voice. Consequently, this meant that the patient felt comfortable, relaxed, less anxious, more positive, less isolated, and more trusted, facilitating patient openness and engagement in their treatment.

*“And she definitely does change her tone of voice, so if I say something that I’m struggling with, you can hear the – I don’t know if it’s sympathy or what, but you can hear that kind of change in her tone of voice that shows that she’s actually properly listened to what I’m saying”. (Patient 1)*

Findings highlighted that reviewing progress across the sessions (via the use of outcome measures or assessing/evaluating goals) indicated some improvement and provided a sense of reassurance and/or achievement, which maintained their motivation and engagement.

All except one of the patients indicated that they felt the practitioner personalised the intervention to their individual needs; they reported feeling understood and treated as a person rather than a number/case; they had the feeling that the practitioner ‘*was on the journey*’ with them.

*“Just the remembered, you know, little bits of details, I never had to repeat myself and I never felt like…sometimes you can feel like you know when people have a lot of clients, patients, whatever you want to call them, that they kind of just one rolls onto the next and it gets a bit mixed up, but I never felt like that, I always felt like that he’d obviously read his notes before he spoke to me and refreshed his memory. But it felt like…yeah, I felt like a person, not a number”. (Patient 5)*

### 5-Process

The sub-domains of planning and engagement provided relevant information about increasing the likelihood of implementation success and uptake of our intervention. Below we describe two key aspects.

- ***Formal planning***

Data from practitioners and service leads highlighted that no formal planning of tasks related to intervention implementation took place in advance. It is hypothesised that the lack of planning around how to implement and monitor each element of a complex intervention could lead to failure and potentially weaken the mechanism of change. Emphasis on clear planning including a) what, who and how the intervention will be implemented and b) monitoring over time, may increase implementation success.

*“So I think the traffic light system is definitely needed to keep it on the agenda. And I think maybe for me, it needs to be like on my agenda with the service manager, when I have my one-to-ones, so she can say, where are you at with that? Are you on amber? When are we going to green? So she can keep it on her radar and it’s brought up at every time or every so often in our supervision session of how well that’s going in regards to implementing those things.” (Service Lead 1A)*

- ***Engagement of appropriate individuals***

There was a shared perception amongst those practitioners interviewed that the lack of engagement of stakeholders such as service leads and team managers in the implementation process had a negative impact. In addition, the lack of clarity about the role of service leads, team managers, practitioners, and other staff from IAPT services (e.g., administrative staff) in the implementation of the intervention and its three different components, appeared to have weakened the contribution to the change mechanisms set out in the ToC. For instance, engagement with the Telephone Recommendations for Services ranged from reading the booklet (no recommendations implemented) to immediately implementing the recommendations identified as most relevant. Examples of implemented recommendations included: addressing safe space and confidentiality within the therapy agreement, allocating time in supervision to reflect on telephone experiences and its challenges, incorporating time into staff induction sessions to discuss uncertainties, and anxieties related to telephone working.

Data suggest that nominating an implementation champion to be responsible for the implementation of the intervention and dedicated to monitoring its progress over time, including identifying and overcoming challenges or resistance from staff, could optimise implementation success.

*“I value the telephone work, so it sort of suits me a bit. But having someone to take responsibility for it ensures that things will get done, ensures that, well hopefully, that someone who volunteers for it, you know, is more likely to action it.” (Service Lead 4)*

*“Maybe just a reminder email or maybe something from whoever organised it, just a little reminder to our managers as something just to remind the staff about that training.” (Practitioner 1)*

### The theory of change for the EQUITy  research

**Fig. 3:**
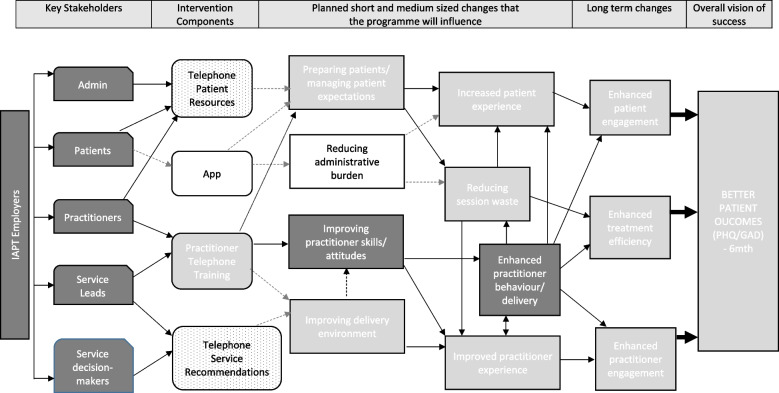
EQUITy Programme: Theory of Change updated following findings from this feasibility study and aimed to be evaluated in a randomised controlled trial Note: White boxes in grey lines indicate elements removed from the intervention prior to the commencement of the feasibility study. Light grey boxes highlight elements that are potentially diluted/uncertain and indicate additional uncertainty/risk. Grey dash lines indicate diluted pathways/linkages. White boxes with a dotted pattern indicate challenges on the implementation of components weakening anticipated linkages. The dotted line represents an additional linkage. Thick black arrows indicate pathways to be assessed by mainly quantitative methods; the rest of the pathways will be mainly assessed by qualitative methods (via a process evaluation)

**Fig. 4:**
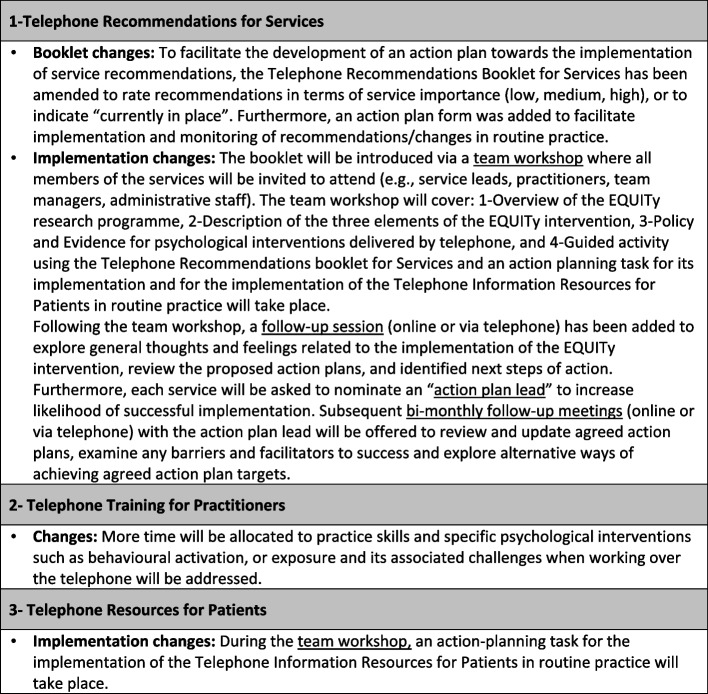
Summary of changes to the EQUITy intervention and implementation strategies following findings from this feasibility study and aimed to be implemented and evaluated in a randomised controlled trial

Following findings from this feasibility study, the ToC depicted in Fig. 2 was interrogated, reviewed, and updated by the research team. After agreement with the IRG and the PSC was confirmed, the ToC was amended and pathways to be assessed by qualitative or quantitative methods were represented in Fig. 3. In addition, changes to the content and the implementation of the EQUITy service quality improvement intervention were identified and reported in Fig. 4. Incorporated changes are aimed at improving understanding, guidance, and support to services. Changes are expected to enhance engagement and strengthen the pathways established to promote change and optimise the service quality improvement of, and engagement with, psychological therapies delivered by telephone. The updated version of the intervention and the ToC will be evaluated as part of a randomised controlled trial.

## Discussion

The assumption that face-to-face systems and processes may simply transfer to a different medium such as the telephone, has hampered service development and delivery in the past [37, 38]. Previous research within the EQUITy research programme has highlighted the factors that are considered to prevent optimisation of psychological guided-self-help interventions delivered by telephone in primary care [6, 7, 26–28]. Drawing on this research, a service quality improvement intervention to increase patient, practitioner, and service engagement with telephone delivery was co-developed with stakeholder informants (patients, practitioners, service leads, team managers, clinical academics, and policy makers) [29]. The MRC framework developed for evaluating the process of complex public health interventions was published in 2015. The MRC framework strongly recommended the use of a theory-driven process and emphasised the importance of intervention implementation evaluations to increase the likelihood of success of complex interventions [21]. This feasibility study research makes important contributions to the literature by detailing challenges and barriers encountered during the implementation phase of a complex intervention that may help to improve engagement in and quality of telephone treatment in mental health services. We applied the CFRI and the ToC towards this end and findings informed requisite changes to our intervention theory and implementation strategies. In addition, we provided four key recommendations that could help to optimise future intervention implementation in stretched and busy mental health services.

### 1-Developing a good understanding of the intervention and its value

Many factors can impede uptake of service quality improvement interventions for telephone working including competing demands on frontline providers, lack of knowledge, skills and resources, and misalignment with service priorities. Therefore, more emphasis should be placed on developing a good understanding of the intervention, identifying its value, exploring resources available, and pinpointing the best way to integrate the intervention within existing procedures to prevent further burden on services. In addition, it is important to overcome any negative beliefs or resistance from staff to prevent implementation failure.

### 2-Increasing engagement from key stakeholders

Findings from our study indicate that a service quality improvement telephone intervention comprised of different components targeting changes at different levels (service, professionals, and patients) requires engagement, commitment, and accountability of different key stakeholders, including service leads, team managers, practitioners, administrative personal, and other staff. Engagement from appropriate individuals could be improved by providing support from leads and using organisational incentives such as goal sharing awards or extrinsic incentives could prove beneficial to increase receptivity and increase engagement of individuals to use the intervention. It is anticipated that high levels of commitment and involvement of leads and managers with the implementation of each of the elements of a complex intervention could enhance the likelihood of a successful implementation. Due to the high turnover of staff in mental health services, it is important to develop strategies to immerse new staff in the awareness and implementation of the intervention, which could ensure sustainability of the intervention over time.

### 3-Clear planning and communication of implementation goals

The lack of time allocated to planning behaviours and implementation tasks, alongside the lack of identification of service quality improvement telephone goals and how to incorporate the intervention in day-to-day work tasks may explain lack of implementation success. Clear planning and communication around implementation goals and what is needed, from whom, and how those would be achieved, are essential to improve the likelihood of a successful outcome. Nominating an implementation lead and identifying champions [39] could be useful in terms of supporting future implementation.

### 4-Formal strategies to monitor implementation progress

Developing models to guide and identify implementation strategies such as audit feedback [40] and implementation performance monitoring could prove fruitful in optimising implementation success and sustainability over time. Formally and effectively monitoring implementation progress and providing feedback to staff on the implementation goals and achievements gradually may help to increase motivation and commitment towards its implementation and could enhance the likelihood of successful implementation. Any implementation progress materials should be user friendly and easily editable to facilitate team communication and participation from different individuals.

### Further reflections on the ToC

The ToC originally developed was in line with a pre-COVID context where the use of telephone to deliver assessment and treatment sessions in NHS Talking Therapies services varied, but many low intensity practitioners (Step 2 care) were delivering approximately 60% of their appointments by telephone [41] without dedicated training [6, 7]. Due to the COVID-19 pandemic, mental health services have had to shift from face-to-face or blended care to entirely remote delivery in a very short time. Although our ToC was reviewed prior to the commencement of the feasibility study to reflect the use of online portals for outcome measure completion and communication with patients (i.e. app removed), there was inevitable variability and some knowledge gaps about how each of the services managed this sudden shift. Findings from this feasibility study highlighted that, in contrast to usual practice pre-COVID, practitioners were mandated to work over the telephone in their day-to-day practice and developed their own skills through experience. Due to increased exposure to remote working because of the COVID pandemic, services, practitioners, and patients became more attuned to this mode of delivery. Evidence from studies published prior to the pandemic did not show interactional differences between psychological therapy delivered face-to-face and by telephone [42]. However, beliefs about telephone being less formal than face-to-face, or being a lower or second best option focused more on reducing costs, were strongly held by patients and professionals. During the pandemic, most services, professionals, and patients were grateful for being able to deliver/receive psychological support during extremely difficult and unprecedented times, influencing their views towards a more positive approach to telephone therapy. Furthermore, findings from this feasibility study identified changes occurring at a service level to meet the needs of practitioners in light of this shift to telephone/online delivery, including the provision of further training, support, and supervision.

Findings from the feasibility study presented in this paper have provided information on individual and group influences on behaviour and the ability that social context, as much as clinical context, has influenced the ToC success of our service quality improvement intervention. Findings from this feasibility study additionally resulted in changes to the content of our EQUITy intervention and its implementation. It is anticipated that changes will increase the likelihood of implementation success for its further evaluation in a randomised controlled trial.

## Conclusions

Concerted attention to evidence-based implementation strategies is needed to reduce the likelihood of implementation failures and optimise success. Implementation strategies should emphasise the value and benefits related to implement an intervention and facilitate awareness and good understanding of what is needed and from whom to implement the intervention. In addition, ensuring engagement and monitoring of progress from key stakeholders is also paramount to improve the likelihood of success and sustainability over time. Understanding implementation challenges for service quality improvement interventions could prove beneficial to increase chances of implementation success.

## Data Availability

The dataset generated and analysed during the current study are not publicly available due to privacy and ethical restrictions (i.e. potential for breach of anonymity); but are available from the corresponding author upon reasonable request.

## Abbreviations

CFIR: Consolidated Framework for Implementation Research
EQUITy: Enhancing the quality of psychological interventions delivered by telephone
IAPT: Improving Access to Psychological Therapies (now called Talking Therapies)
IRG: Implementation Reference Group
MRC: Medical Research Council
NIHR: National Institute of Health Research
PSC: Programme Steering Committee
ToC: Theory of Change
